# In Situ Analyses of Surface-Layer Composition of CxNy Thin Films Using Methods Based on Penning Ionization Processes—Introductory Investigations

**DOI:** 10.3390/ma14247812

**Published:** 2021-12-17

**Authors:** Galina Grigorian, Izabela Konkol, Adam Cenian

**Affiliations:** 1 Physics Department, St. Petersburg State University, 199034 St. Petersburg, Russia; galgr2@rambler.ru; 2 Physical Aspects of Eco Energy Department, The Szewalski Institute of Fluid-Flow Machinery, Polish Academy of Sciences, 80-231 Gdansk, Poland; izabela.konkol@imp.gda.pl

**Keywords:** carbonitride, thin films, film diagnostics, Penning ionization, electron energy distribution function

## Abstract

Carbon nitride materials have received much attention due to their excellent tribological, mechanical and optical properties. It was found that these qualities depend on the N/C ratio; therefore, the possibility to control it in situ in the sputtered film is of high importance. The plasma-electron spectroscopy method based on the Penning ionization process analysis is developed here to control this ratio in CN_x_ films produced by plasma-sputtering in a pulsed-periodic regime of glow discharge. The electron energy distribution function is determined by the means of a single Langmuir probe placed in the center of the discharge tube. The mixture N_2_:CH_4_:He was used in the process of sputtering. The applied concentrations of CH_4_ varied in the range of 2–8%, and He concentration was 80–90%. The gas pressure in the discharge tube used for sputtering varied between 1 and 10 Torr, and the current was between 10 and 50 mA. It was shown that the proposed method enables the extraction of information on the composition of the surface layer of the investigated film and the development of an on-line inspection, without extracting the film from the sputtering chamber.

## 1. Introduction

The interest in carbon nitride materials has arisen due to their expected/computed hardness (comparable to diamond) [1] and high thermal conductivity (exceeding 100 W m^−1^ K^−1^) [2]. Later, part of this interest moved to the carbonitride thin films, due to their excellent tribological, mechanical and optical properties [3,4,5,6]. The graphitic carbon nitride (g-CN) has attracted much attention from the community in developing inorganic semiconductor photoelectrodes (for water splitting, production of hydrogen [7], conversion of CO_2_ into valuable products [8], and synthesis of fine chemicals [9,10,11,12]) due to its merits, namely an appropriate bandgap, abundant composition elements, good thermal stability and non-toxicity [13]. However, the scope of application for this material spans beyond photocatalysis, for example, to optoelectronics, energy and membrane separation, etc. [13,14].

Carbon-nitride (CN) films have been produced using various methods, such as radio frequency (RF) sputtering [15], ion vapor deposition [16], ion beam assisted deposition [17], ion plating [18], pulsed laser deposition [19], reactive magnetron sputtering [4], plasma-enhanced chemical vapor deposition (PECVD) [20], glow discharge sputtering [21] and high-power impulse magnetron sputtering (HiPIMS) [22]. It was observed that the film structure and characteristics depend strongly on the nitrogen-to-carbon (N/C) ratio and temperature—see e.g., [23]. For example, it was found that at low temperatures, amorphous films grow, while at higher temperatures, a graphitic structure forms when nitrogen content is low (below 5%), and a fullerene-like structure forms with higher nitrogen content.

Various diagnostics were used to determine the main CN film characteristics. The profilometry and absorbance spectrum fitting (ASF) methods were successfully used to determine thickness and optical band gaps of amorphous carbon nitride thin films [24]. The effect of nitrogen incorporation on the bonding states and growth kinetics of the deposited films have been investigated using Fourier transform infrared spectroscopy (FTIR), Raman spectroscopy and X-ray photoelectron spectroscopy [20]. In addition, optical properties were determined by UV (ultraviolet) spectroscopy measurement. The band gap was found to decrease with the increase in nitrogen concentration. The solid-state 13C NMR (Nuclear Magnetic Resonance) spectroscopy was used to determine chemical bonding [5].

Since 1990, more attention has been paid towards in situ thin film diagnostics in order to control the films’ fabrication during manufacture [25,26,27]. Sakurai et al. [26] observed fabrication of Cu(In,Ga)Se_2_ (CIGS) thin films using the pyrometer and the spectroscopic light-scattering (SLS) techniques. The film deposition process, including its speed, thickness, compositional ratios, surface roughness and precipitation of Cu-rich phases, has been monitored in situ. White light reflectometry (WLR) was used in situ for non-destructive monitoring of the deposition technique of Cu-Zn-Sn-S thin films by thermal vacuum evaporation (PVD) [27]. In addition, the Langmuir probe diagnostics of the microwave ECR (Electron Cyclotron Resonance) plasma (2.45 GHz, 1.5 kW) were used for the diagnostics of the thin film deposition process [28]. This allowed the determination of charged particle density, electron temperature, plasma potential and floating potential, which helped to control the deposition process. Irimiciuc et al. [29] developed the Langmuir probe method further as a real-time in situ diagnostic tool for laser ablation and pulsed laser deposition. The Langmuir probe technique led to several discoveries, such as multiple peak distribution, selective acceleration during expansion, plume splitting, plasma turbulences and fluctuations.

Here, a plasma-electron spectroscopy (PES) method is described, which may allow in situ control of C_x_N_y_ thin film composition. The interaction of plasma active species (mainly charged species and metastables) with carbonitride films leads to a partial destruction of the surface layer and emission of some atoms and molecules into the bulk plasma. These species, if excited, can be observed in the spectra. Detailed analyses of these spectra may enable the extraction of information on composition of the surface layer of the film, although the quality of spectral information may inhibit the analysis, due to a low concentration of the mentioned species.

In order to increase the sensitivity, plasma-electron spectroscopy (PES) is applied here. The technique has already been proposed for an investigation of plasma processes in low-pressure gas discharges [30,31,32]. The PES method is based on the analysis of the electron energy distribution function (EEDF), especially for electrons produced in chemo-ionization processes, e.g., due to Penning ionization.

R* + X → R + X^+^ + e, (1)

where R* is the excited atom, and X is the species in the ground state. The largest contribution to the process (1) has metastables, R* = R_m_, as their concentration in plasma is higher than other excited species. Especially interesting are processes where R_m_ is the helium metastable state, as the ionization (1) of all species in the plasma is energetically possible. Nonetheless, for Ne ionization (though Ne is not present in the plasma mixture considered here) energy E(R_m_) is too low.

The electrons produced in the process (1) will have energy equal ε_x_ ≈ E_m_ – E_i_, where E_m_ is the energy of the excited atom, and E_i_ is the ionization energy for the species X. Both E_m_ and E_i_ are atomic or molecular constants, and therefore the electron energy, ε_x_, is specific for each species and does not depend on the conditions of the experiment or on the presence of other species. Therefore, a detailed analysis is possible, which allows the identification of peaks appearing in the EEDF spectra (corresponding to ε_x_) and of existing species in bulk plasma.

The identification of the peaks is completely hidden in EEDF spectra of the stationary direct current (DC) plasma, where a strong acceleration in the DC electric field leads to electron heating and the appearance of high-energy electron flows. The situation (scenery) changes when observing plasma in the discharge decay stage (e.g., afterglow); the influence of electron acceleration on EEDF is hardly observed, as the electric field is negligible. Only then (for negligible E) registration of EEDF peaks, due to the processes (1), as well as the identification of X species, is possible.

The PES method was applied successfully for the determination of the impurities concentration in helium discharge (see e.g., [33]). It is expected that PES enables information on the composition of the surface layer of films by better identification of the species appearing in the bulk plasma.

The aim of the paper is to extract information from the N/C composition, which is crucial for the quality of the investigated film, and to develop a novel in situ (on-line) control method without extracting the film from the sputtering chamber. The EEDF will be investigated, taking into account Penning ionization with the participation of helium excited states (He(2^3^S) and He(2^1^S)) and the species that appear during plasma C_x_N_y_ film interactions. The EEDF spectrum is registered by means of a single electrode (Langmuir probe) placed in the center of the discharge tube.

## 2. Experimental Methods

**Figure 1:**
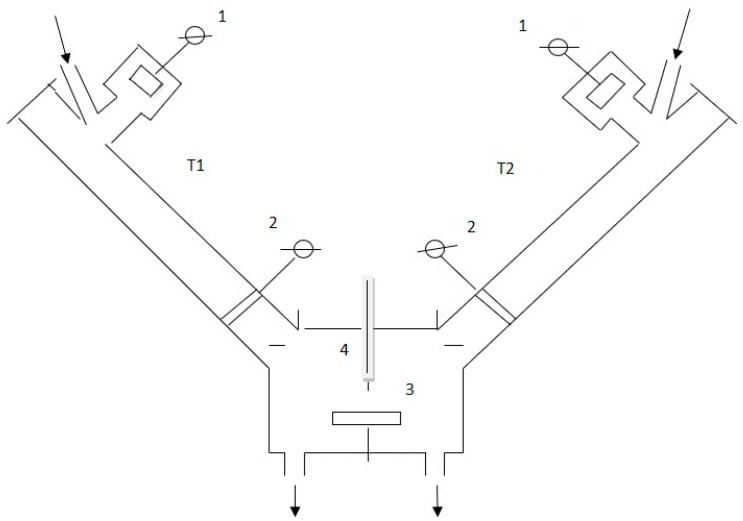
Scheme of experimental setup: T1 and T2—discharge tubes, 1 and 2—electrodes, 3—target with thin film, 4—Langmuir probe; arrows show the gas inlet and outlet; two similar and independent discharges operate between both electrodes 1 and 2.

First, the CN_x_ film was produced by the plasma-sputtering method in a pulsed-periodic regime of glow discharge in the experimental setup shown in Figure 1. Later, the gas mixture of the sputtering phase was removed, and spectrally purified helium (He) was introduced into the discharge reactor to perform the PES analysis. The frequency of the discharge during both phases was in the range of 2–3 kHz, with a pulse duration at about 90 ms. The discharge was generated in a twin tube setup (Figure 1) with a radius of 3 cm. The setup with twin tubes leads to a higher rate of film production. The polarization of the electrodes can be changed without visible influence on film-growth characteristics.

### 2.1. CN_x_ Production Phase

The CN_x_ film was produced from the method described in Reference [21]. The mixture N_2_:CH_4_:He was used in the process of sputtering. The applied concentrations of CH_4_ varied in the range of 2–8%, and the He concentration was 80–90%. The gas pressure in the discharge tube used for sputtering was varied between 1 and 10 Torr, and the current was between 10 and 50 mA. The film was grown on the silicon tiles 10–15 cm in diameter. The composition of the films obtained (ratio of nitrogen to carbon, N/C) was determined by using the energy-dispersion Roentgen (X-ray) (Oxford Instruments Inc., Abingdon, UK) microanalyses and detector Oxford INCA X-act (Oxford Instruments Inc., Abingdon, UK). The N/C ratio of the films varied across a wide range, depending on discharge conditions.

### 2.2. PES In Situ Analysis

The discharge was generated in the same setup after removing the gas mixture of the sputtering phase and by refilling it with spectrally purified He. The gas pressure was varied between 0.5 and 2 Torr. The Langmuir probe method [34] was used in order to determine the spectrum of EEDF in the afterglow phase of the discharge. A molybdenum probe (0.045 mm in radius) was placed in the center of the discharge tube, 2 cm above the tile covered, and with 1–2 mm of carbonitride film. 

The earthed cathode of the discharge was used as the base electrode for probe measurements.

### 2.3. Langmuir Probe Data Analysis

As mentioned above, the registration of the electron energy spectrum was performed under afterglow conditions, i.e., 100–200 μs after the current pulse. For EEDF determination, the probe-current-modulation method (the time resolution was 6 μs) was used. In this method, the second derivative of the probe current-voltage characteristic, d^2^i_e_/du^2^, is determined, which is related to the electron energy distribution function through the Druyvesteyn relation [31,34,35,36].

(2) $$f(\xi )|{}_{\xi =eV}=\frac{2{\left(2m/e\right)}^{1/2}}{e^{2}S}d^{2}i_{e}(V)/dV^{2},$$

where *S* is the probe area, *V* is the probe potential with respect to the plasma potential, *I_e_* is the electron probe current, and *f(ε)* is the normalized electron energy distribution function:

(3) $$\int_{0}^{\infty }f_{0}(\xi )\sqrt{\xi }d\xi =1.$$

Here, the second derivative of the probe current is determined using a method of current modulation (e.g., [30,32,33] and references herein). In this method, a small amplitude AC voltage signal is applied to the probe circuit (differential signal), and the harmonics of the probe current are registered—see also [32,37,38,39,40]. In our case, ∆*V*(t) = *a*●cos(ωt) signal was applied, i.e., the second harmonic of the probe current (with frequency 2*ω*) under the condition of small ‘*a*’ is proportional (through Taylor expansion) to the second derivative of the probe current—$i_{2\omega }\approx \frac{a^{2}}{4}i^{\prime \prime }$ [9]. So, by registering the second harmonic of the probe current, one gets information on the second derivative of the probe current and, consecutively, EEDF.

## 3. Results and Discussion

**Figure 2:**
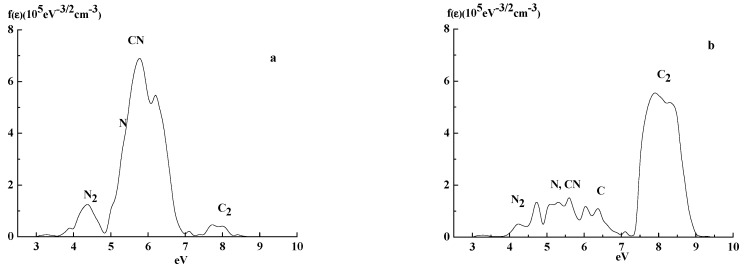
EEDF in a pure He-discharge afterglow period (τ = 150 μs after pulse end) for energy 3–10 eV, *p* = 1 Torr, I = 80 mA for N/C ratios (**a**) 0.63 and (**b**) 0.18.

A typical EEDF was determined using the Langmuir probe, with structures in regions, where the Penning ionization processes considered here appear, as is shown in Figure 2. One sees the complex structure of an electron energy spectrum. The ionization processes with the participation of He metastables leads to the production of fast electrons and non-monotonic structures of the spectra.

It should be underlined that Penning ionization processes appear also in pure He plasma, when two excited particles collide [30,31]

He(2^3^S_1_) + He(2^3^S_1_) → He_2_^+^ + e ΔE = 14.4 eV He(2^1^S_1_) + He(2^3^S_1_) → He_2_^+^ + e ΔE = 15.4 eV He(2^1^S_1_) + He(2^1^S_1_) → He_2_^+^ + e ΔE = 16.2 eV (4)

**Figure 3:**
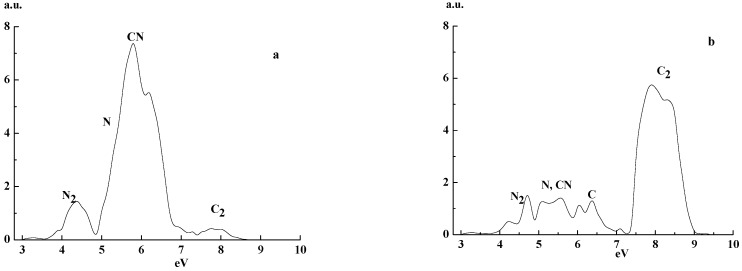
EEDF in a pure He-discharge afterglow period (τ = 200 μs after pulse end) for energy 3 up to 10 eV, *p* = 1 Torr, I = 80 mA for N/C ratios (**a**) 0.63 and (**b**) 0.18.

However, these processes lead to the appearance of relatively intensive peaks in the range of 14–16 eV of EEDF. These peaks do not interfere with the structures observed below 10 eV, presented in Figure 2 and Figure 3.

There is also a continuous spectrum in helium plasma, which results from the interaction of two He metastables

He(2^3^S_1_) + He(2^3^S_1_) → He^+^ + He(1^1^S) + e.

This process generates electrons with energy in the range of up to 14.5 eV. Moreover, quasi-continuous spectrum results from so-called second-kind (superelastic) collisions of electrons with helium metastables, as described by Capitelli et al. [35]. These processes could not strongly interfere with the EEDF peaks related to the type of reactions considered here—process (1), as they generate a quasi-continuous spectrum.

In addition, there is also another way to proceed. For example, one might subtract the spectrum for pure He plasma from the one recorded for the process of thin film deposition. As the deposition process does not influence plasma parameters, the proposed subtraction enables determination of EEDF structures related to the deposition process.

Figure 2a presents the electron energy spectrum registered by the Langmuir probe immersed in discharge plasma to be used for the control of the C/N ratio (0.63) in a C_x_N_y_ film surface. The peak in the range 6 eV corresponds to the electrons generated in the processes.

He(2^3^S_1_) + CN → He + CN^+^ + e (5.6 eV), (5)

He(2^1^S_1_) + CN → He + CN^+^ + e (6.3 eV). (6)

In addition to the main maximum, related to Penning ionization with participation of CN molecules, the electron energy spectrum shows a series of other peaks, which correspond to Penning ionization processes with participation of N_2_, C_2_ molecules, as well as the atomic species N and C. All these species appear in the bulk plasma due to an interaction with CxNy films.

Presently, very few data exist that correspond to elementary processes related to film-growth and its composition. It is assumed that the basic species (precursors) generated in the initial stage of discharge during sputtering in N_2_–CH_4_ plasma are the CN molecules and nitrogen atoms (see e.g., [41,42,43]). Their concentrations determine the speed of film generation, but its characteristics (described by the N/C ratio) depend on the CN/N ratio in the gas phase. From the spectra in Figure 2a, one can deduce that the main products of film (with a high N/C ratio) destruction by an active species of He-plasma are the CN molecules.

The electron energy spectrum in Figure 2b presents data for the case with carbonitride film with a small N/C ratio (0.18); it is very different from the previous one. One observes a strong decay with a maximum in the region of 6 eV, which indicates a decrease in CN concentration. An increase in maximum in the region of 8 eV corresponds to electrons generated during Penning ionization with the participation of carbon molecules:

He(2^3^S_1_) + C_2_ → He + C_2_^+^ + e (7.9 eV), (7)

He(2^1^S_1_) + C_2_ → He + C_2_^+^ + e (8.6 eV). (8)

**Figure 4:**
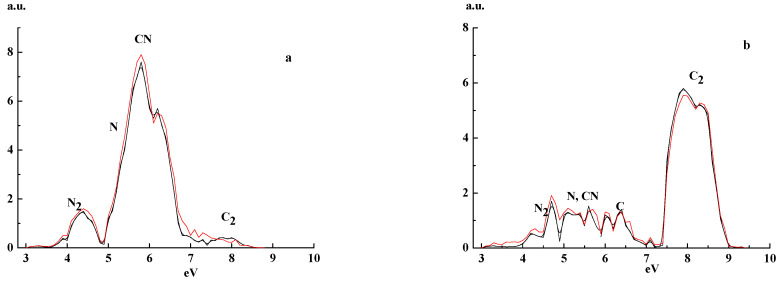
EEDF in a pure He-discharge afterglow period (τ = 200 μs—black curves, and 300 μs—red curves, after pulse end) for energy 3 up to 10 eV, *p* = 1 Torr, I = 80 mA for N/C ratios (**a**) 0.63 and (**b**) 0.18.

Figure 3 presents electron energy spectra measured τ = 200 μs after the discharge pulse end. Comparing Figure 2, Figure 3 and Figure 4, one deduces that the main features of the spectra do not depend on a time span (150–300 μs) after the discharge pulse end.

In conclusion, the Langmuir probe characteristics, measured during thin CxNy film production in the process of glow-discharge plasma-sputtering, enable the deduction of EEDF in plasma above the film. Furthermore, it was found that the structure of the determined EEDF is strongly related to the N/C ratio. This fact could be used in situ to control the composition of thin CxNy film, without extracting the film from the sputtering chamber.

Moreover, it is well known that the main characteristics of carbonitride films, including mechanical, optical, electrophysical, etc., are determined by the relative concentration of atoms with orbital hybridization sp^3^ and sp^2^ as well as the N/C ratio [44,45,46,47]. In addition, as the N/C ratio declines, the unique characteristics of these films are less pronounced.

## 4. Conclusions

The possibility of controlling the N/C ratio in the sputtered film is of high importance. Nowadays, the analysis of film composition is usually performed using mass spectrometry or X-ray microanalysis. It was shown here that plasma-electron spectroscopy (PES) facilitates obtaining information on the composition of the surface layer of the investigated film. Moreover, the method allows for the development of on-line inspection, without extracting the film from the sputtering chamber.

Here, the preliminary results of such methodology are presented and discussed. The method should be optimized in relation to current, the gas pressure in the discharge tube, registration time (after the discharge pulse end) and probe geometry. It can be expected that such optimization can increase the volume of data that could be extracted from the spectra.

## Data Availability

Not applicable.

## References

[1] Liu A.Y., Cohen M.L. (1990). Structural properties and electronic structure of low compressibility materials: B-Si3N4 and hypothetical b-C3N4. Phys. Rev. B.

[2] Morelli D.T., Heremans J.P. (2002). Thermal conductivity of germanium, silicon, and carbon nitrides. Appl. Phys. Lett..

[3] Khurshudov A., Kato K., Sawada D. (1996). Tribological and mechanical properties of carbon nitride thin coating prepared by ion-beam-assisted deposition. Tribol. Lett..

[4] Broitman E., Hellgren N., Wänstrand O., Johansson M.P., Berlind T., Sjöström H., Sundgren J.E., Larsson M., Hultman L. (2001). Mechanical and tribological properties of CNx films deposited by reactive magnetron sputtering. Wear.

[5] Gammon W.J., Malyarenko D.I., Kraft O., Hoatson G.L., Reilly A.C., Holloway B.C. (2002). Hard and elastic amorphous carbon nitride thin films studied by 13C nuclear magnetic resonance spectroscopy. Phys. Rev. B.

[6] Hoh H.Y., Zhang Y., Zhong Y.L., Bao Q. (2021). Harnessing the Potential of Graphitic Carbon Nitride for Optoelectronic Applications. Adv. Opt. Mater..

[7] Wang X., Maeda K., Thomas A., Takanabe K., Xin G., Carlsson J.M., Domen K., Antonietti M. (2009). A metal-free polymeric photocatalyst for hydrogen production from water under visible light. Nat. Mater..

[8] Mazzanti S., Cao S., ten Brummelhuis K., Völkel A., Khamrai J., Sharapa D.I., Youk S., Heil T., Tarakina N.V., Strauss V. (2021). Allorganic Z-scheme photoreduction of CO2 with water as the donor of electrons and protons. Appl. Catal. B.

[9] Ghosh I., Khamrai J., Savateev A., Shlapakov N., Antonietti M., König B. (2019). Organic semiconductor photocatalyst can bifunctionalize arenes and heteroarenes. Science.

[10] Savateev A., Antonietti M. (2018). Heterogeneous Organocatalysis for Photoredox Chemistry. ACS Catal..

[11] Khamrai J., Ghosh I., Savateev A., Antonietti M., König B. (2020). Photo-Ni-Dual-Catalytic C(sp2)-C(sp3) Cross-Coupling Reactions with Mesoporous Graphitic Carbon Nitride as a Heterogeneous Organic Semiconductor Photocatalyst. ACS Catal..

[12] Mazzanti S., Kurpil B., Pieber B., Antonietti M., Savateev A. (2020). Dichloromethylation of enones by carbon nitride photocatalysis. Nat. Commun..

[13] Xiong W., Huang F., Zhang R.-Q. (2020). Recent developments in carbon nitride based films for photoelectrochemical water splitting. Sustain. Energy Fuels.

[14] Kumru B., Antonietti M. (2020). Colloidal properties of the metal-free semiconductor graphitic carbon nitride. Adv. Colloid Interface Sci..

[15] Cuomo J.J., Leary P.A., Yu D., Reuter W., Frisch M. (1979). Reactive sputtering of carbon and carbide targets in nitrogen. J. Vac. Sci. Technol..

[16] Fujimoto F., Ogata K. (1993). Formation of carbon nitride films by means of ion assisted dynamic mixing (ivd) method. Jpn. J. Appl. Phys..

[17] Bousetta A., Lu M., Bensaoula A., Schultz A. (1994). Formation of carbon nitride films on Si(100) substrates by electron cyclotron resonance plasma assisted vapor deposition. Appl. Phys. Lett..

[18] Taki Y., Kitagawa T., Takai O. (1997). Shielded arc ion plating and structural characterization of amorphous carbon nitride thin films. Thin Solid Film..

[19] Yap Y.K., Kida S., Aoyama T., Mori Y., Sasaki T. (1998). Influence of negative dc bias voltage on structural transformation of carbon nitride at 600 _C. Appl. Phys. Lett..

[20] Rusop M., Abdullah S., Podder J., Soga T., Jimbo T. (2006). Optical and structural properties of nitrogenated diamond-like carbon films prepared by r.f. PECVD. Surf. Rev. Lett..

[21] Grigorian G.M., Kochetov I.V. (2013). Preparation of carbonitride films in the active and afterglow phases of a glow discharge. Plasma Phys. Rep..

[22] Cometto O., Dennett C.A., Tsang S.H., Short M.P., Teo E.H.T. (2018). A thermal study of amorphous and textured carbon and carbon nitride thin films via transient grating spectroscopy. Carbon.

[23] Hellgren N., Johansson M.P., Broitman E., Hultman L., Sundgren J.-E. (1999). Role of nitrogen in the formation of hard and elastic CNx thin films by reactive magnetron sputtering. Phys. Rev. B.

[24] Escobar-Alarcón L., Arrieta A., Camps E., Muhl S., Rodil S., Vigueras-Santiago E. (2007). An alternative procedure for the determination of the optical band gap and thickness of amorphous carbon nitride thin films. Appl. Surf. Sci..

[25] Herman I.P. (1996). Optical Diagnostics for Thin Film Processing.

[26] Sakurai K., Scheer R., Kaufmann C.A., Yamada A., Baba T., Kimura Y., Matsubara K., Fons P., Nakanishi H., Niki S. (2004). In situ diagnostic methods for thin-film fabrication: Utilization of heat radiation and light scattering. Prog. Photovolt Res. Appl..

[27] van Duren S., Levcenco S., Kretzschmar S., Just J., Unold T. (2019). Investigation of reflectometry for in situ process monitoring and characterization of co-evaporated and stacked Cu-Zn-Sn-S based thin films, Journal of Alloys and Compounds. J. Alloys Compd..

[28] Singh S.B., Chand N., Patil D.S. (2008). Langmuir probe diagnostics of microwave electron cyclotron resonance (ECR) plasma. Vacuum.

[29] Irimiciuc S.A., Chertopalov S., Lancok J., Craciun V. (2021). Langmuir Probe Technique for Plasma Characterization during Pulsed Laser Deposition Process. Coatings.

[30] Kolokolov N.B., Blagoev A.B. (1993). Processes of Ionization and Quenching with Creation of Fast Electrons. Usp. Fiz. Nauk.

[31] Kolokolov N.B., Kudryavtsev A.A., Blagoev A.B. (1994). Interaction Processes with Creation of Fast Electrons in the Low Temperature Plasma. Phys. Scr..

[32] Blagoev A.B., Papov T., Pilosoff N., Ogoyski A., Rusinov I. (2006). Investigation of the interactions of long-lived excited atoms in the afterglow of gas discharge plasma. J. Phys. Conf. Ser..

[33] Sheverev V.A., Khromov N.A., Kojiro D.R. (2002). Penning ionization electron spectroscopy in glow discharge: Another dimension for gas chromatography detectors. Anal. Chem..

[34] Chung P.M.-H. (1975). Electric Probes in Stationary and Flowing Plasmas: Theory and Application.

[35] Capitelli M., Celiberto R., Colonna G., Esposito F., Gorse C., Hassouni K., Laricchiuta A., Longo S. (2016). Fundamental Aspects of Plasma Chemical Physics: Kinetics.

[36] Raizer Y.P. (1991). Gas. Discharge Physics.

[37] Kolokolov N.B., Smimov B.M. (1985). Chemoinization Processes in Low Temperature Plasma. Chemistry of the Plasma.

[38] Blagoev A.B., Kagan Y.M., Kolokolov N.B., Lyagushchenko R.I. (1975). Finite amplitude distortion of the electron distribution measured by probe current modulation. Zh. Tekh. Fiz.

[39] Bang J.Y., Kim A., Chung C.W. (2010). Improved measurement method for electron energy distribution functions with high accuracy and reliability. Phys. Plasmas.

[40] Lee H.C., Kim A., Moon S.Y. (2011). Observation of pressure gradient and related flow rate effect on the plasma parameters in plasma processing reactor. Phys. Plasmas.

[41] Vlcek J., Rusnak K., Hajek V., Martinu L. (2000). New Approach to Understanding the Reactive Magnetron Sputtering of Hard Carbon Nitride Films. Diam. Relat. Mater..

[42] Popov C., Plass M.F., Bergmaier A., Kulisch W. (1999). Synthesis of Carbon Nitride Films by LowPower Inductively Coupled Plasma-Activated Transport Reactions from a Solid Carbon Source. Appl. Phys..

[43] Pereira J., Massereau-Guilbaud V., Geraud-Grenier I., Plain A. (2005). CH and CN Radical Contribution in the Particle Formation Generated in a Radio-Frequency CH4/N2Plasma. Plasma Process. Polym..

[44] Sato G., Samano E.C., Macherro R., Farias M.H., Cota-Araiza L. (2001). Study of composition and bonding character of CNx films. Appl. Surf. Sci..

[45] Cappelli E., Trucchi D.M., Kaciulis S., Orlando S., Znaza A., Mezzi A. (2011). Effect of deposition temperature on chemical composition and electronic properties of amorphous carbon nitride (a-CNx) thin films grown by plasma assisted pulsed laser deposition. Thin Solid Film..

[46] Popov C., Zambov L.M., Plass M.F., Kulisch W. (2000). Optical, electrical and mechanical properties of nitrogen-rich carbon nitride films deposited by inductively coupled plasma chemical vapor deposition. Thin Solid Film..

[47] Grigorian G.M., Cenian A. (2011). Formation and Excitation of CN Molecules in He–CO–N2–O2 Discharge Plasmas. Plasma Chem. Plasma Process..

